# Implementing public health nursing training for Ireland's National Healthy Childhood Programme

**DOI:** 10.1111/phn.13049

**Published:** 2022-01-17

**Authors:** Helen Mulcahy, Carmel Brennan, Anne Pardy, Brenda McCormack, Julie Heslin

**Affiliations:** ^1^ Doctor of Nursing College lecturer School of Nursing and Midwifery at University College Cork Cork Ireland; ^2^ Community Health Programme Manager Health Service Executive National Healthy Childhood Programme Tullamore Ireland; ^3^ Programme Lead in Health Service Executive Nurture Programme Health Service Executive Tullamore Ireland; ^4^ Project support Nurture Programme Tullamore Ireland; ^5^ Nurture programme Tullamore Ireland

**Keywords:** child health, implementation science, public health nursing, skills‐based training

## Abstract

Public health nurses (PHNs) in Ireland provide preventative child health. An evidence‐based National Healthy Childhood Program (NHCP) has been in development since 2016. The final program implementation, including training all PHNs coincided with the Covid‐19 pandemic.

**Objective:**

To describe implementation and evaluation of a blended training program for PHNs

**Design:**

The evaluation used quantitative and qualitative methods underpinned by an implementation science framework to assess the training program. The three‐phase blended training was led by a Training and Resources implementation team. Data from a national cohort of PHNs (*n* = 1671) who completed training were descriptively analysed.

**Results:**

The majority of PHNs completed a suite of four online units (phase 1), as well as self‐directed and asynchronous content in phase 2. Results of phase 2 indicated it met participant needs in terms of knowledge but outstanding needs in terms of skills remained. Phase 3 (a modified Face to Face Clinical Skills Review) was completed by 1671 PHNs over a 5‐month period in 2020. Evaluation was very positive in terms of organisation and usefulness for practice.

**Conclusions:**

Despite challenges the NHCP training implementation goals were met. A well‐designed blended learning training program met service delivery imperatives and PHN needs.

## 1 BACKGROUND

Ireland has a long history of public health nurses (PHNs) providing a preventive child health service. This universal service is supported by national policy and is enshrined in legislation. A comprehensive review of the service was carried out in early 2000 and commenced again from 2015. This universal Child Health Screening Surveillance and Health Promotion (CHSSHP) program was formalised in 1999 (Midland Health Board, 1999) and updated by Denyer et al. (2005) and is known in the system as Best Health for Children.

The CHSSHP was embedded in practice with a program of national Continuous Professional Development (CPD) training. This was provided to all PHNs and Community Medical Doctors (CMDs) who were the main health care professionals (HCP) delivering this program. The CPD took the form of training manuals and Face‐to‐Face delivery of a suite of nine modules. The quality and cost effectiveness of such programs are a very important consideration in any jurisdiction, no more so than with our United Kingdom (UK) neighbours in the National Health Service (NHS) (Banning & Stafford, 2008).

Cost and technological advancements have driven CPD delivery globally in the past two decades. Mlambo et al. (2021) described the mandatory versus non‐mandatory approaches to CPD which led to a rationale to examine how nurses perceive and experience it in a recent meta synthesis. Their findings will be discussed later but they suggest that top‐down requirements are key drivers. Regulatory bodies like the Nursing and Midwifery Board of Ireland (NMBI) or professional bodies such as the Royal College of Nursing (RCN) in the UK have explicitly stated commitments to lifelong learning. Furthermore, health services have specific needs for ensuring that all relevant staff are prepared to deliver new programs. In Ireland there is a culture of community nursing staff engaging with such mandatory training and of it being monitored locally by PHN management. The UK's Healthy Child Program (Royal College of Paediatrics and Child Health [RCPCH], 2021) Healthy Child Program which is like the Irish NHCP has an online learning program accessible by registration with a suite of 76 training modules of relevance to health visitors, specialist PHNs and others involved in the delivery of the universal preventative child health service delivery. The platform used is called e‐Learning for Healthcare (e‐LfH) and is a Health Education England Program, in partnership with Professional Bodies and the NHS (2021a). In the United States of America (USA) the Center for Disease Control's CDC TRAIN (2021) is the external learning management system (ELMS) for CPD for health professionals. This facility has an extensive catalogue of CPD training for HCPs caring for children including web based, blended, and many other formats.

According to Heslin (2017) updated child health CPD training in Ireland was long overdue and implementation demanded the development of a strong training framework. Continuing with CHSSHP CPD after the initial years did suffer from funding cuts and logistical challenges in terms of training delivery (HSE, 2018a). Consequently, there was a strong appetite for long promised National Healthy Childhood Program (NHCP) (Health Service Executive, 2021a; HSE, 2018a, 2018b) and the CPD plans which would accompany it.

The revised NHCP service incorporates child health screening and surveillance, health promotion and vaccination, and includes the most recent evidence on such topics as infant mental health, breastfeeding, nutrition. The program is supportive of the new national strategies on maternity (Department of Health, 2016) and early years services (Government of Ireland, 2018). It was acknowledged that implementation of the NHCP across the health service would be a complex process. There are nine Community Healthcare Organizations (CHO) which deliver community health services in Ireland. Nursing services in each Local health Office (LHO) area within a CHO is led by a Director of Public Health Nursing (DPHN) and the latest workforce figures indicate a staff of 1536 whole time equivalent PHNs in employment at the end of April 2021 (HSE, 2021b). There was a culture of each CHO and sometimes LHO organising and delivering their own Face‐to‐Face training, but this approach would not ensure a seamless and timely transition to the new NHCP. Updating the training for service‐providers, including PHNs, was essential to its successful roll‐out.

The implementation process was informed by implementation science principles (Centre for Effective Services [CES], 2021) as part of an overall quality improvement initiative for universal child health services, known as the *Nurture Program–Infant Health and Wellbeing*. The Program aimed to support the strategic reform of universal health and wellbeing services for infants and their families, made possible through a grant from the Atlantic Philanthropies. This integrated program of work built on new evidence (collated and evaluated through the NHCP Evidence reviews) in developing updated professional resources and public‐facing information platforms through an organisational structure of six enthusiastic multidisciplinary teams. Application of implementation science principles to the process involved a series of methodologies, including extensive parent and practitioner engagement and user testing of outputs in service settings. Luyckx et al. (2019) summarises some of the key differences between implementation research and basic research. Of key importance is comprehensive stakeholder involvement in the planning, implementation, and post research phases to ensure success and long‐term sustainability.

In the planning phase (Luyckx et al., 2019), the Training and Resources team which included the authors of this paper (chaired by JH) as well as key stakeholders identified the subject areas required for child health practice. The knowledge and skills requirement of each subject and the best fit for online or in‐person training were also examined (O'Meara & Quinlan, 2015). The team provided oversight to the development and delivery of a suite of online training units to be hosted on HSELand, the HSE's online learning and development portal (OLDP). A total of 18 modules were developed, focusing on learning objectives relevant to child health practice needs. The development of each module involved a multi‐step process including initial development of core content and collation of supporting resources by subject matter experts, central coordination by a clinically led project team, user testing by frontline practitioners and final quality assurance of content and functionality by further subject matter experts. Using robust pedagogical underpinnings, a skills training framework matrix (led by HM) was developed and refined. It was initially envisaged that this would be a two‐day face – to – face Clinical Skills Review training workshop for all frontline PHNs in Ireland.

The new NHCP was due to be implemented in March 2020, including practice changes and revised timing of core child health assessments. Implementation was supported by a new clinical practice manual for PHNs and a comprehensive nationally standardised child health record. This final stage of implementation followed the earlier completion of updated print materials for parents and an online child health information website www.mychild.ie. Unfortunately, the final implementation of the NHCP, including delivery of training program to all PHNs in the state, coincided with the global Covid‐19 pandemic. A national lockdown commenced in Ireland on the 13th of March 2020 with the need to pivot the training plans to a revised blended learning package. This paper represents an overview and evaluation of the revised evidence‐based blended training program to a national cohort of PHNs.

## 2 THE REVISED BLENDED LEARNING PROGRAM

The revised blended learning package had three main phases for implementation. **Phase 1** was the requirement for PHNs to complete 4 online e‐learning units, namely: Primary Child Health Assessment; Undertaking the 3‐month assessment; Undertaking the 9–11 month assessment; Undertaking the 21–24 month and 46–48 month assessment.

**Phase 2** contained a suite of short asynchronous informational videos by subject matter experts. These videos provided updated evidence, guidance, and skills demonstrations, relevant to specific PHN knowledge and assessment of clinical skills, in line with the new practice requirement of the NHCP. The seven areas where either PHN practices would be changing or where there was professional disquiet about changes were: (a) Undescended Testes (UDT); (b) Head Circumference; (c) Jaundice; (d) Primitive Reflexes; (e) Developmental Dysplasia of the Hip; (f) Partnership with Parents and relationship building; and (g) Key messages for PHN practice at the 9–11 month visit. Additionally, the self‐directed training package, envisaged to take two hours to complete, contained a link to an online questionnaire on Survey Monkey™ to assess whether they had sufficient knowledge on the seven topics covered on the pre‐recorded subject expert videos and if additional skills for practice were required.

**Phase 3** was a 2 h Clinical Skills Review workshop delivered by two facilitators, conducted under stringent Covid‐19 restrictions. A team of facilitators (*n* = 9) was convened and attended training (led by HM). A training pack was designed (HM), agreed and distributed to ensure a standardised approach by facilitators to the CSR training. The program was coordinated centrally by two of the authors of this paper (AP and BMcC) and their colleagues. They ensured that each participant received URL links to Phase 2 (online videos and questionnaire), as well as a date (within a two‐week period), to attend the CSR workshop, and guidance on Covid‐19 compliance at the planned venues. Feedback on phase 2 analysis was provided to each facilitator for each CHO phase 3 workshop to enhance their preparation for the training. Participants at phase 3 completed their evaluation questionnaire prior to leaving the venue.

## 3 METHODS

The evaluation design was underpinned by an implementation science framework and used quantitative and qualitative methods. These included participant e‐learning activity and completion data from phase 1, as well as participant attendance and self‐reports from phases 2 and 3. Evaluation was undertaken using the realist evaluation approach (CES, 2021). This method recognizes the complexity of context as well as other factors in gauging the success of real‐world interventions. It is concerned with “what works, in what circumstances, and for whom?” (CES, 2021 p.1). For the team this meant that they were acutely aware that process was as important as outcome, as there were implementation challenges in the different CHOs. Compliant with Implementation Science principles (Luyckx et al., 2019), stakeholders from the Training and Resources Group remained involved in this phase to ensure smooth implementation of the training plans. The three‐phase blended training was managed by the Nurture NHCP office, and all elements relating to plans, process and outcomes were regularly collated and reviewed.

### 3.1 Sample

Data from a national cohort of PHNs (*n* = 1671) who completed the blended training program were descriptively analysed.

### 3.2 Measures

The HSE NHCP implementation group captured e‐learning activity and completion data by professional group using a built‐in dashboard on HSEland. These completion rates were extracted for this paper. Scheduling at venues and attendance data for the CSR training were collected and maintained in the HSE by the authors of this paper (CB, AP, and BMcC).

Data on self‐directed pre‐learning self‐assessed knowledge and skills was collected by a newly developed questionnaire hosted on Survey Monkey. This anonymised self‐directed pre‐learning questionnaire (SDPLQ) had simple dichotomous questions which measured whether participants had sufficient knowledge and need for additional skills on the seven topics covered on the accompanying pre‐recorded expert videos. Options for open responses were provided. The purpose of this SDPLQ was firstly to encourage participant self‐reflection on knowledge and skills in relation to seven specific topics. Secondly, it would provide the clinical facilitators with data on skills deficits or areas of concern for discussion, specific to each CHO and related to the NHCP roll‐out, in advance of each scheduled phase 3 CSR session.

A new questionnaire was also designed to capture participant data on completion of the phase 3 Clinical Skills Review. The variables of interest in the Clinical Skills Review Questionnaire (CSREQ) were effectiveness of the pre‐CSR self‐directed learning; expectations of participation met; utility for practice; organisation of the training and overall assessment of the CSR training. These questions were essentially seeking data on knowledge change and satisfaction with training. Although satisfaction surveys are commonly used, Willis et al. (2016) caution that they are not simply a matter of examining the gap between expectations and experiences. To strengthen this survey method a variety of three‐ and five‐point Likert scales were used to collect data. Additionally, open questions were provided to allow participants to expand on responses and provide greater insight to the quantitative data.

This training program evaluation did not fall within the remit of the health service executive ethics committees. However, participants were verbally informed at each education session of the purpose of the evaluation and that evaluation would be shared widely. Additionally, the authors ensured no identifying data were collected and participants had an opportunity to choose, complete, and submit the questionnaires in private. A decision was taken in the preparation of this paper to report some data in aggregate form to minimise any possibility of CHO identification from divergent results.

### 3.3 Analytic strategy

In line with implementation science principles the authors were concerned with the process, implementation, and outcome of this national project. Logistical data were provided to illustrate the delivery of the NHCP. Quantitative data from participants were descriptively analysed. Qualitative data were content analysed and divergent narrative quotes used where appropriate to provide insight to the quantitative results. These narrative quotes were labelled R (for Respondent) and the corresponding questionnaire number from the data file.

## 4 RESULTS

The implementation of the blended learning program was achieved for all 9 CHOs in Ireland. It was coordinated from the Nurture NHCP office and ran over 5 months. Booking for the CSR commenced in July 2020 and concluded in December 2020. A “mop up” of the blended learning training program ran from April to June 2021 to facilitate PHNs staff (*n* = 100) who for a variety of reasons were unable to attend in 2020. This coincided with a national cyber‐attack on the HSE and because all IT data systems were inaccessible at this time no evaluation from attendees of “mop‐up” training are included in this paper.

### 4.1 Completion of phase 1 online e‐learning units

In phase 1 of the blended program PHNs were requested to complete 4 online e‐learning units prior to booking and participation in phases 2 and 3. Table 1 below illustrates the completion rates as of 31.03.21.

**TABLE 1:** Uptake of online e‐learning units hosted by HSEland

Name of unit	*N*
Primary child health assessment	1783
Undertaking the 3‐month assessment	1708
Undertaking the 9–11 month assessment	1673
Undertaking the 21–24 month and 46–48 month assessment	1602

This table indicates that figures for those who completed the training in three of these four units exceeded the numbers booked to attend subsequent training (*n* = 1671). A possible explanation will be provided in the discussion.

### 4.2 Pre‐CSR training

**FIGURE 1:**
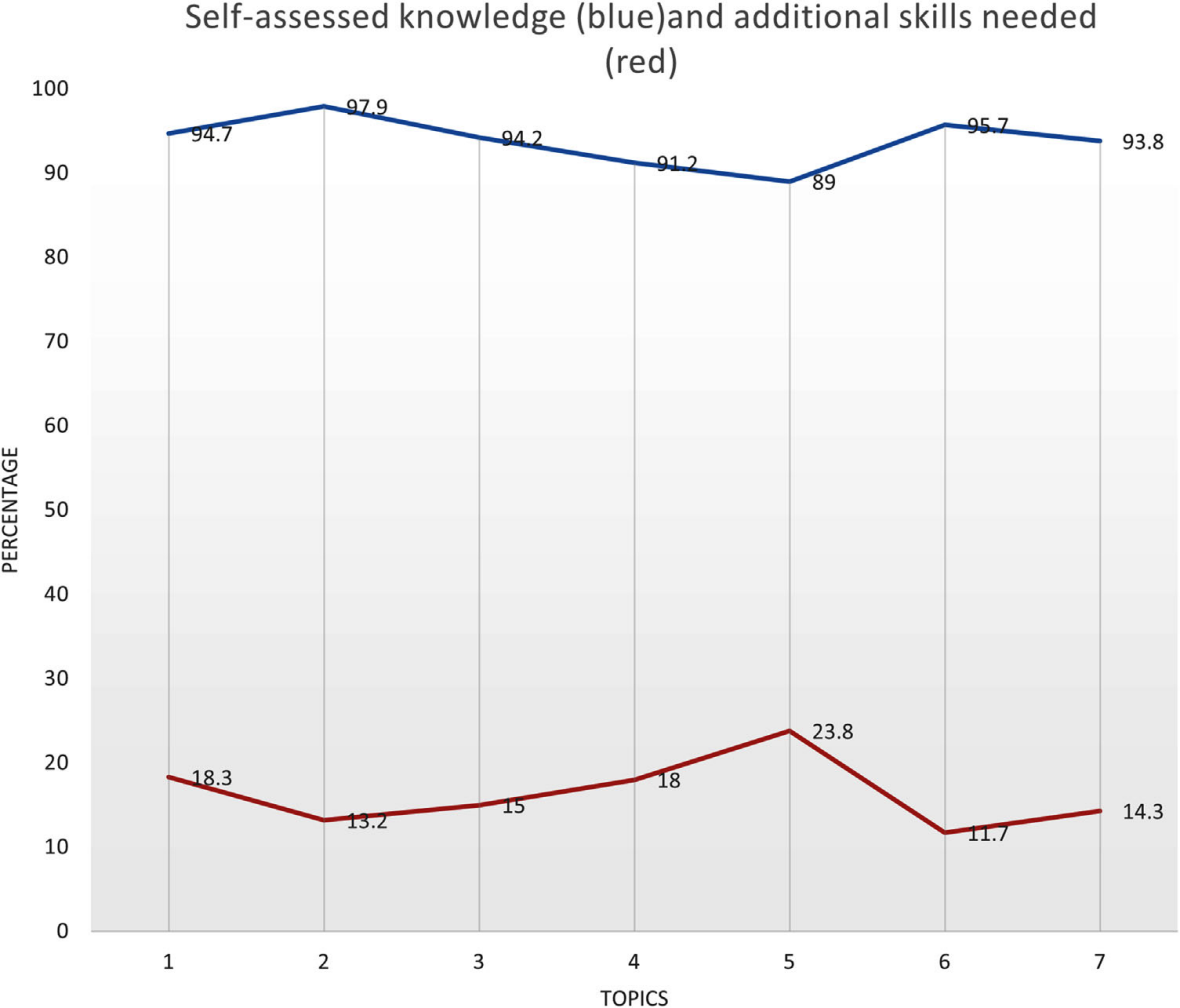
Self assessed knowledge and additional skills needed. Seven topic areas covered in phase 2 as follows: 1, Undescended Testes; 2, Head Circumference; 3, Neonatal Jaundice; 4, Primitive Reflexes; 5, Developmental Dysplasia of Hip; 6, Partnership with parents; 7. 9–11 month check. Blue data line = self‐assessed knowledge. Red data line = additional skills needed [Colour figure can be viewed at wileyonlinelibrary.com]

For phase 2 the SDPLQ was completed by 1234 PHNs. Although this data are available to the authors by each of the individual CHO areas and is more variable, it cannot be reported for this paper at this level for HSE confidentiality reasons. Nevertheless, total data are presented for self‐assessed knowledge and skills needed in Figure 1 below.

Figure 1 shows that participants scored highly on self‐assessed knowledge across the seven topic areas (Mean 93.6%, range 89%–97%). The process in phase 2 had encouraged participants to reflect and self‐assess their knowledge and skills after viewing pre‐recorded expert videos on seven areas where changes in practice were required. Participants were also required to self‐assess whether additional skills were still needed. Figure 1 shows that while these scores are much lower (mean 16.6%, range 11.7%–23.8%), it provides the rationale for phase 3 of the blended training. DDH was the topic with the lowest self‐assessed knowledge and highest additional skills needed scores.

### 4.3 Post CSR training evaluation

The CSREQ was returned by 1610 of the 1671 participants who attended the CSR, representing a 96% response rate. The results are presented in Tables 2 and 3 below.

**TABLE 2:** Effectiveness of SDL, overall assessment and event organisation

Rating	Excellent 5		4		3		2		Unsatisfactory 1		Not Recorded	
Question	F	%	F	%	F	%	F	%	F	%	F	%
Effectiveness of “self‐directed” learning	735	45.7%	656	40.7%	144	8.9%	17	1.1%	13	0.8%	45	2.8%
Overall Assessment of the CSR program	1053	65.4%	444	27.6%	57	3.5%	3	0.2%	0	0	53	3.3%
Organisation of the CSR event	1104	68.5%	372	23.1%	64	4%	5	0.3%	7	0.4%	58	3.6%

Abbreviations: F, frequency; %, percentage.

**TABLE 3:** Topics covered, learning outcomes, expectations, utility for practice

Response	Yes		No		Somewhat		Not Recorded	
Question	F	%	F	%	F	%	F	%
Were topics in SDL addressed in CSR?	1352	84%	9	0.6%	51	3.2%	198	12.3%
Did the CSR program achieve its aims and learning outcomes?	1568	97.4%	7	0.4%	Not asked		35	2.2%
Did the knowledge and information gained on the program meet expectations?	1521	94.5%	2	0.1%	64	4.0%	23	1.4%
Will the knowledge and information gained be useful in my work?	1348	83.7%	85	5.3%	14	0.9%	163	10.1%

Abbrevaitions: F, frequency, % = percentage).

Table 2 presents results in relation to participant rating of: effectiveness of the self‐directed learning in phase 2; their overall assessment of the 2‐h Face‐to‐Face CSR program: and overall assessment in relation to how it was organised.

Well over 80% of participants selected high ratings of four or five. In terms of organisation of the event this was especially reassuring for the HSE as the CSR was delivered during a pandemic lockdown and fears had been expressed about complying with Covid 19 regulations. One participant stated that the organisers “*did very well under the current covid restrictions*” (R 70) whereas a more negative view was a participant who expressed disappointment in being “*obliged to attend the training during restrictions*” (R 948). There were many positive comments about having the CSR opportunity because “*person to person meetings to discuss practices are always very beneficial*” (R 39). In terms of self‐directed learning one participant felt the process was “*a little rushed ‐ difficulty getting video done in work time*” (R 1354). Some stated the approach used meant they would lose the opportunity for interactive group learning.

Table 3 illustrates responses to questions about: alignment of the seven key topics in self‐directed learning phase 2 with phase 3; assessment of the program achieving its aim and learning outcomes; meeting participant expectations; and whether learning gained would be useful for practice.

Results were very positive overall. In particular, 97% of participants perceived that the CSR had achieved its aim and learning outcomes. Figures for “not recorded” may be accounted for by the reported information communication technology (ICT) problems experienced by some who had attended Phase 3 but had not been able to access the URL links in phase 2. A high percentage (94.5%) indicated that the CSR had met their expectations and slightly less (84%) in terms of knowledge and information being useful for practice.

Positive participant comments included focus on the “*practical skills session ‐ hips, reflexes*” (R 108) and the opportunity for “*clarification on issues in relation to baby / child examination*” (R 336). Many participants commented on teaching strategies used in phase 3 such as *practical demonstrations* (R 1404)*, scenarios* (R 330) *discussions* (R 1417)*, role play* (R 1437). Facilitators were praised as “(they) *were excellent, very knowledgeable and able to highlight the new changes*” (R 343). Negative comments were very few but related to a desire for “*more time*” (R367, R 468, R 934) and more *“social interaction”* (R 730).

## 5 DISCUSSION

Although the blended learning element of this training for PHNs took place over 5 months the entire project has been in place for over 4 years with a strong implementation science inspired governance structure. A program position paper (O'Meara & Quinlan, 2015) initially recommended a blended training which balanced the advantages and disadvantages of differing e‐learning and workshop approaches underpinned by pedagogy to enhance learning. The entire project was supported by national strategy (HSE, 2018a) and included all relevant stakeholders. This ensured expectations in terms of program planning and delivery were constantly reviewed and managed. Careful and responsive governance is illustrated by the way in which the original blended plan comprising a suite of online e‐learning modules followed by a 2 day Face‐to‐Face clinical skills training had to pivot (like many educational/or training programs globally), to a re‐designed blended program within a couple of months. At this time in Ireland Covid‐19 restrictions permitted only essential training face to face under stringent conditions. Designing teaching and learning strategy for effective implementation was an integral feature of the revised process. Ousey and Roberts (2013) recommend the development of non‐traditional study packages to maximise uptake. According to Uprichard (2020) there is much to recommend flexibility in location and method of delivery in achieving effective outcomes for CPD. Although Uprichard's (2020) paper was focused on e‐learning there was also an exploration of the benefits of asynchronous learning for HCPs.

The results in the current study of the asynchronous self‐directed pre‐CSR in phase 2 demonstrate the benefit for participants in term of reinforcing knowledge acquisition and allowing subsequent training to focus specifically on skills. The approach adopted is supported by Cappi et al. (2019) who recommend that blended learning providers need to pay attention in the design and implementation phases of blended modules. Their guidance to assess participants' needs was very important to the current team so that content in terms of training would meet knowledge and skills needs and properly meet program learning outcomes.

Results from completion of the initial online training units on HSEland in phase 1 suggest full engagement by Irish PHNs. However, it must be borne in mind that data did not differentiate the grade of PHN. The authors of this paper are aware of student PHNs, academic PHNs and PHN specialists and management who undertook these four units, and thus were included in completion rates. Nevertheless, knowing that there are 2655 nurses on the public health division of the register maintained by the NMBI (2019), and that there were 1536 whole time equivalent PHNs in employment at the end of April 2021, the authors have confidence in the full reach of this required blended program. This contrasts with results from a large sample of PHNs and community RGNs studied by the Irish Nurses and Midwives organisation (INMO, 2013) in Ireland. This found that almost three quarters of respondents were facilitated to attend mandatory training required for nursing in the community. However much has changed in terms of training delivery in eight years. Digital technology does not have all the answers though, and our findings do have implications for future data management in the e‐learning portal, in terms of capturing useful attendance data. Nonetheless, in other jurisdictions such as in the UK only high‐level numbers such as registered users, and sessions completed etc. are available for e‐learning attendance (NHS Health Education England, 2021b).

Results indicated that the self‐directed learning phase 2 was effective. They revealed a difference between self‐assessed knowledge on specific topics and additional skills needed for practice. However, this was expected and was the rationale for having 3 phases. Differences in terms of rating knowledge and skills acquisition was not surprising to the authors and is supported by evidence (Steven et al., 2018). Elshami et al. (2021) found that undergraduate level interactivity between teacher and learner is required for an authentic learning experience. There was anecdotal as well as unpublished evidence (O'Meara & Quinlan, 2015) since the beginning of this project that PHNs had a strong interest in skills acquisition and had fears that the development of online and blended training would negate their belief in the higher value of Face‐to‐Face training. Steven et al. (2018) opined that online learning is often seen as a panacea for all in times of austerity and in the rush to achieve homogenous outputs. The authors and their colleagues were very clear on the value of the process in achieving final training goals and learning for the future.

Steven et al.’s (2018) study although small did highlight the value of CPD workshops for a small sample of health visitors and other multidisciplinary members (*n* = 21) in the north of England. Workshops were viewed by participants as informative and provided them greater insight regarding roles, services and processes contributing to enhancement of practice in terms of supporting families and effective referrals post workshop attendance. In a mixed methods study (Fotheringham, 2013) teaching strategies such as facilitator feedback adopted in workshops was considered vital to development skill and judgment.

In terms of utility for practice the content of the blended program focused on seven key topics that emerged as a relevant to practice, from a robust iterative process. Findings support taking this approach, and this aligns with evidence (Ousey & Roberts, 2013) that qualified nurses want to avail of training that develops knowledge and skills on specific areas relevant to their work. Steven et al. (2018) recommended that initiative developers need to nurture social capital as well as paying attention to the context and mechanisms to enhance attendance and engagement along with application in subsequent practice. Mlambo et al. (2021) in an up‐to‐date meta synthesis of 25 qualitative studies found that perceived impact on practice was a core value of nurses in relation to CPD. Ultimately there is a strong motivation to enhance knowledge and skills. The current study supports the findings of Mlambo et al. (2021).

The findings relating to the organisation and delivery were very positive and suggest that the blended program was realistic, attainable, and relevant and thus align with Mlambo et al.’s (2021) recommendations for CPD. The authors were mindful of McCutcheon et al.’s (2015) review finding that the blended learning approach to teaching clinical skills (albeit in undergraduate education) is characterised by a lack of evidence on implementation. The authors have provided much descriptive detail about the implementation, but future evaluation will be refined in line with realist evaluations (CES, 2021; Luyckx et al., 2019).

In conclusion, this paper provided detail in relation the planning and delivery of large‐scale program of blended training attended by PHNs, to support the implementation of an updated preventative child health model. The evaluation was very positive in terms of organisation, content, delivery, and relevance for PHN practice.

### 5.1 Limitations of the study

A strength of this program is its underpinning by implementation science. This ensured stakeholder contribution at all phases of the process. Findings illustrate very high attendance at training, positive engagement, and relevance for PHN practice. The high response rate to the final evaluation is a strength of this study. However, the study is limited by newly developed questionnaires which have not been assessed for reliability and validity. More detailed qualitative analysis was beyond the scope of this paper but is being used by the authors to inform on‐going planning. Measuring satisfaction is acknowledged as an inherent weakness generally (Willis et al., 2016) and thus of this study as well. However, future projects as part of the NHCP will include more objective outcome measures.

### 5.2 Recommendations for future research

Participants were surveyed immediately prior to, and on the day of attendance at the Clinical Skills Review which means training content recall was very fresh. It would be useful to assess participants at one year or longer to reassess knowledge, skills, and application of learning to practice. Future research should include more rigorous evaluation of learning outcomes as recommended by Cappi et al. (2019).

### 5.3 Implications for public health nursing

PHNs globally are required to maintain ongoing competence and CPD is of importance to regulators, service managers, educators, and others. At times programs under which PHN practice undergo major evidence‐based changes which require accelerated training delivery for all practitioners. Therefore, the detail provided here of utilising implementation science principles in the delivery and evaluation of a blended learning program will be of interest to PHNs globally.

## ETHICS APPROVAL STATEMENT

This service evaluation does not fall under the remit of a Health Service Executive research ethics committee in Ireland.

## Data Availability

Research data are not shared.
